# Limb saving surgery for Ewing’s sarcoma of the distal tibia: a case report

**DOI:** 10.1186/s12885-018-4372-z

**Published:** 2018-05-02

**Authors:** Naoki Mizoshiri, Toshiharu Shirai, Ryu Terauchi, Shinji Tsuchida, Yuki Mori, Yusei Katsuyama, Daichi Hayashi, Yoshinobu Oka, Toshikazu Kubo

**Affiliations:** Department of Orthopaedics, Graduate School of Medical Science, Kyoto Prefectural University of Medicine, Kamigyo-ku, Kyoto, Kyoto, 602-8566 Japan

**Keywords:** Ewing sarcoma, Distal end of tibia reconstruction, Bone transport method, Iodine coating technique

## Abstract

**Background:**

Ewing’s sarcoma is a primary malignant tumor of bone occurring mostly in childhood. Few effective reconstruction techniques are available after wide resection of Ewing’s sarcoma at the distal end of the tibia. Reconstruction after wide resection is especially difficult in children, as it is necessary to consider the growth and activity of the lower limbs.

**Case presentation:**

A 12-year-old Japanese boy had presented with right lower leg pain at age 8 years. Imaging examination showed a bone tumor accompanied by a large extra-skeletal mass in the distal part of his tibia. The tumor was histologically diagnosed as Ewing’s sarcoma. The patient received chemotherapy, followed by wide resection. Reconstruction consisted of a bone transport method involving external fixation of Taylor Spatial Frame. To prevent infection after surgery, the external fixation pin was coated with iodine. One year after surgery, the patient showed poor consolidation of bone, so iliac bone transplantation was performed on the extended bones and docking site of the distal tibia. After 20 months, tibia formation was good. Three years after surgery, there was no evidence of tumor recurrence or metastases; bone fusion was good, and he was able to run.

**Conclusions:**

The bone transport method is an effective surgical method of reconstruction after wide resection of a bone tumor at the distal end of the tibia, if a pin can be inserted into the distal bone fragment. Coating external fixation pins with iodine may prevent postoperative infection.

## Background

Ewing’s sarcoma is a primary malignant tumor of bone, consisting of proliferating undifferentiated small round cells. Ewing’s sarcoma is the third most common malignant bone tumor, after osteosarcoma and chondrosarcoma, occurring primarily in young people under age 20 years. Its most frequent location is the diaphysis of long bones, especially the femur, tibia, humerus, and pelvis. The 10-year survival rate of patients with Ewing’s sarcoma is 57% [1]. Ewing’s sarcoma responds better to chemotherapy than other primary sarcomas of bone. Effective chemotherapy can reduce surgical margins and make limb preservation procedures possible [2]. Surgical removal of malignant bone tumors in the distal tibia is relatively difficult, as this site contains less subcutaneous tissue than the knee and hip joints. Furthermore, the presence of nerves and blood vessels at this site makes securing safe surgical margins difficult. Thus, amputation or radiotherapy may be the only curative treatment for patients with tumors of the distal tibia [3, 4].

Even if chemotherapy followed by resection is effective in patients with Ewing’s sarcoma of the distal tibia, few effective reconstruction methods are currently available.

Reconstruction after wide resection is especially difficult in children, as it is necessary to consider the growth and activity of the lower limbs.

## Case presentation

**Fig. 1:**
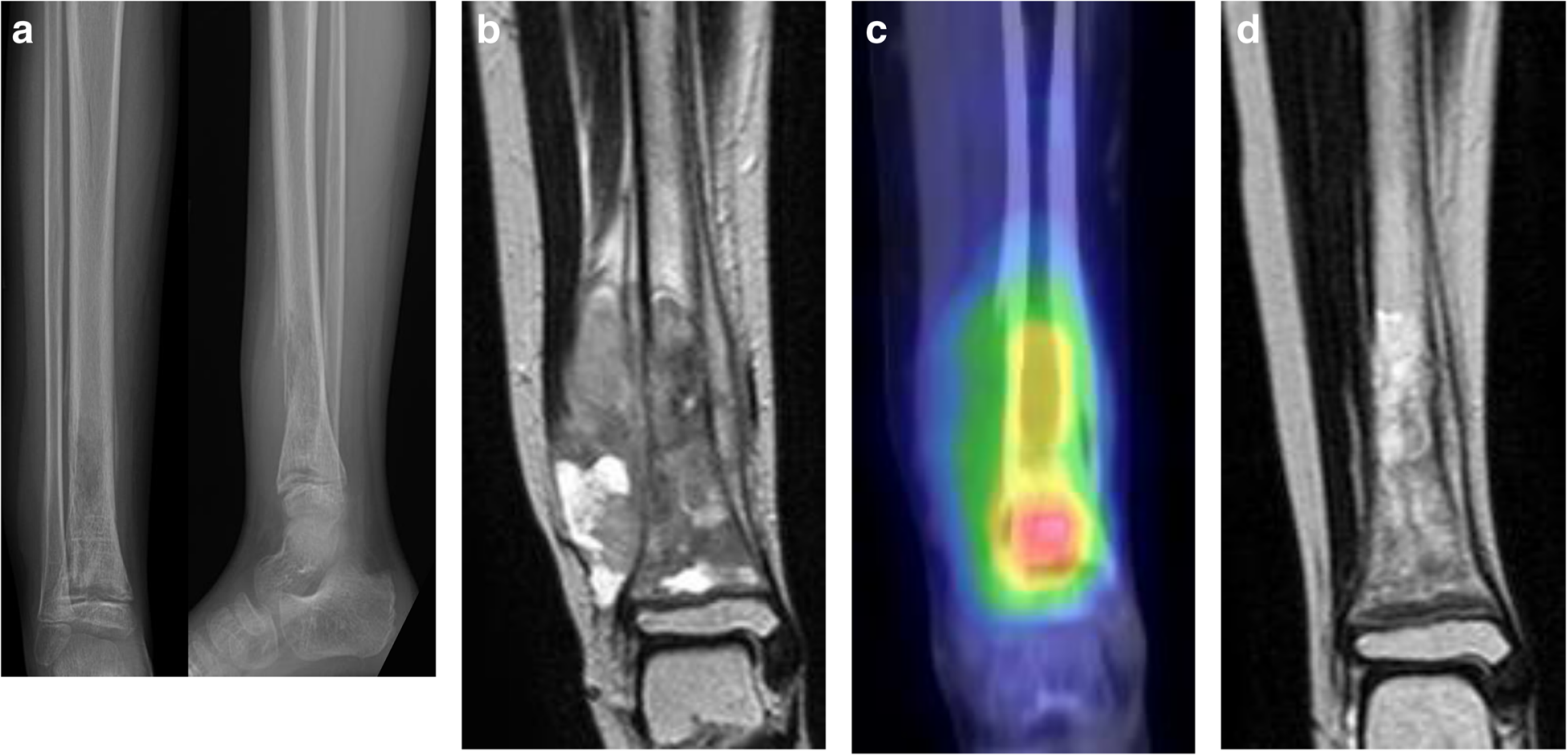
**a** X-ray of the lower leg showing periosteal response, and translucent distal tibia. **b** MRI coronal T2 weighted image showing massive extra-skeletal mass at the anterior distal metaphysis of tibia. **c** Thallium scan, showing high accumulation at the distal metaphysis of tibia. **d** MRI coronal T2 weighted image following chemotherapy revealing reduction in the extra-skeletal mass

A 12-year-old Japanese boy originally presented with right lower leg pain at age 8 years. At that time, his right lower leg showed swelling, was tender, and sensitive to heat. Blood tests showed that the concentrations of lactate dehydrogenase (LDH) and alkaline phosphatase (ALP) were very high, but he was negative for C-reactive protein (CRP). X-rays showed a periosteal response and bone translucency at the distal metaphysis of his right tibia (Fig. 1a). Magnetic resonance imaging (MRI) showed an extra-skeletal mass anterior to the distal metaphysis of the tibia (Fig. 1b). A thallium scan showed high accumulation in early phase and no wash out appearance in delayed phase, with no metastatic lesions (Fig. 1c). Histological examination of an incision biopsy sample showed diffuse proliferation of cytoplasmically poor heterotypic cells positive for EWS-FLI1, resulting in a diagnosis of Ewing’s sarcoma. The patient was administered 3cycles of preoperative chemotherapy, consisting of vincristine (2 mg/m^2^) for 1 day, doxorubicin (37.5 mg/m^2^) for 2 days, and cyclophosphamide (1.2 g/m^2^) (VDC) for 1 day, alternating with ifosfamide (1.8 g/m^2^) and etoposide (100 mg/m^2^) (IE) for 5 days [5, 6].

**Fig. 2:**
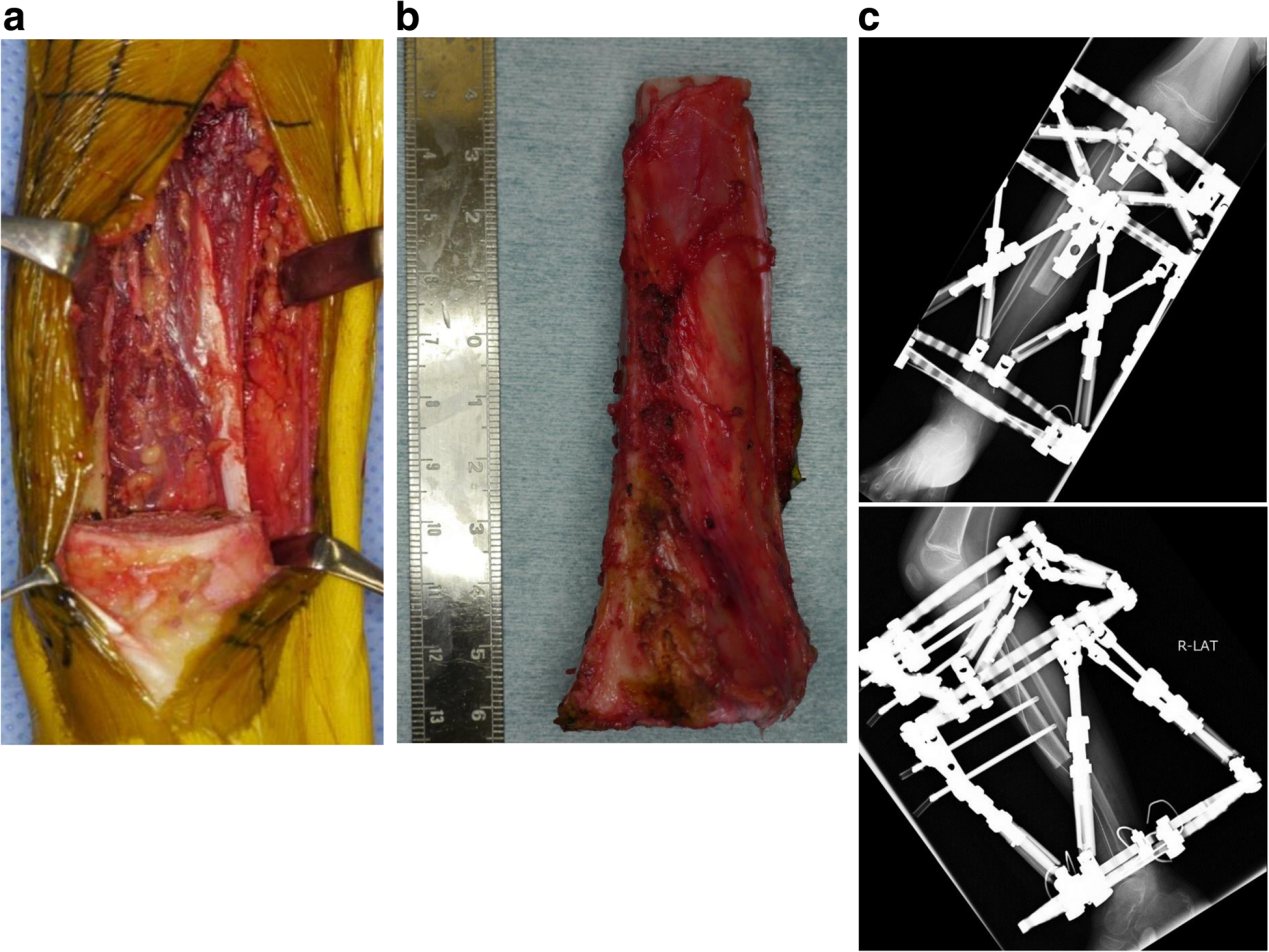
**a** After wide excision, no tumor remained. **b** The excised bone fragment was about 95 mm. **c** X-ray following reconstruction, showing Taylor Spatial Frame for bone transportation

**Fig. 3:**
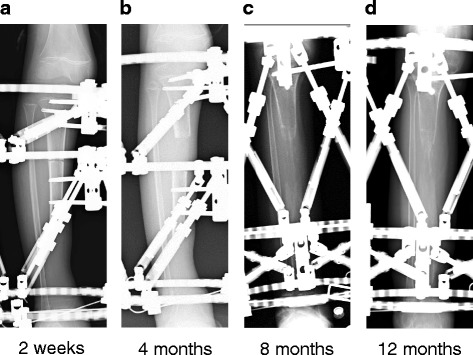
**a** X-ray 2-weeks post-surgery before starting bone transport showing bone fragment was at same site as immediately after surgery. **b** At 4-months, and after starting bone transport, the bone fragment was located in the center of the bone defect **c** At 8-months, morphogenesis of the bone trunk was observed, with the bone fragment in contact with the distal end of the tibia. **d** At 12-months, reformation of the bone trunk was noticeable, but fusion failure with the distal end of the tibia and the bone fragment was observed

**Fig. 4:**
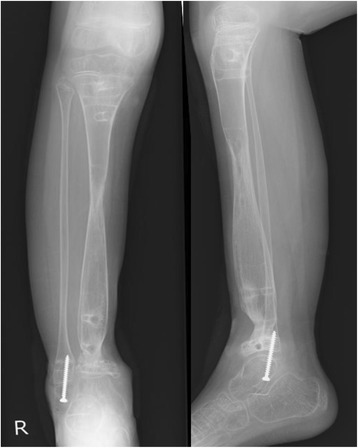
At 1 year 8-months after wide resection, surgery of the distal epiphyseal line closure of the fibula had been performed and tibial bone formation was good

Because MRI after preoperative chemotherapy showed a significant reduction in the size of the extra-skeletal mass (Fig. 1d), surgery was performed with wide excision. Osteotomy of the proximal tibia was performed, with a 30 mm margin from the lesion edge. Osteotomy of the distal tibia was also performed distal to the epiphyseal line. Tumor bone fragments 95 mm in length were excised (Fig. 2a, b). The margins were negative. Further tibial osteotomy was performed at a position 60 mm proximal to the proximal resection edge to move a fragment, followed by transport of the bone fragment using a Taylor Spatial Frame. To prevent postoperative infection, pins coated with iodine were used (Fig. 2c). The percent necrosis of the specimen was estimated to be 100%. Beginning 2 weeks after surgery, the bone fragment was moved 1 mm per day. Three weeks later, the patient was started on post-operative chemotherapy. Bone fragment movement was evaluated every 2 weeks (Fig. 3a-d). Although a foot ring is usually used to prevent ankle equinus deformity during bone transport, a short leg brace was used for this patient. One year after surgery, the patient showed poor consolidation of bone and failure of bone union at the docking site in the distal tibia. Therefore, iliac bone transplantation was performed on the extended bones and docking site of the distal tibia. Twenty months later, tibial bone formation was good, but a tendency toward ankle varus deformity due to growth of the fibula was observed. Distal epiphyseal line closure of the fibula was therefore performed (Fig. 4). Three years after the original operation, there was no evidence of tumor recurrence or metastases. Furthermore, bone fusion was good, he was able to run and his Musculoskeletal Tumor Society (MSTS) score [7] was 100%.

## Discussion and conclusions

Ewing’s sarcoma, a type of malignant bone tumor, occurs more frequently in individuals aged < 20 years than in older individuals. Patients with these tumors have a 10-year survival rate of 57% and poor prognosis. However, patients < 40 years of age with high grade Ewing’s sarcoma have a good prognosis if the tumor is completely resected surgically [8]. Unfortunately, complete surgical resection is likely to result in postoperative dysfunction. The patient described in this report was an 8-year-old boy with Ewing’s sarcoma in the distal end of the tibia. Because chemotherapy resulted in considerable reduction of the extra-skeletal mass, the patient was expected to have a good prognosis after complete resection. However, reconstruction after wide resection of this tumor was difficult, primarily because the distal tibia is anatomically adjacent to the neurovascular bundle and tendons. Thus, limb amputation would likely optimize postoperative function, while minimizing tumor recurrence and metastasis [9, 10].

Advances in neoadjuvant chemotherapy can reduce resection margins, while preserving the limb. Methods of major reconstruction after wide resection of malignant bone include allografts, treated-bone autografts, vascularized fibula grafts and endoprosthetic replacement. Allografts can be physiologically reconstructed, resulting in good postoperative results, but adjuvant treatment increases complications [11]. Use of allografts in Japan is difficult, because a bone bank system has not yet been established. Treated-bone autografts have been associated with good prognosis and postoperative function [3], but bone strength is necessary. Vascularized fibula grafts are likely to result in bone fusion at an early stage. Furthermore, these grafts can be covered by soft tissue after wide resection, with good postoperative limb function expected. However, the fibula is thinner than the distal tibia, increasing the risk of fracture of transplanted bone. In addition, this surgical procedure is complicated and the operation time long [4, 12, 13]. Endoprosthetic replacement results in instant stability and quick recovery of ankle function, allowing load walking to start early. However, instability, misalignment, and difficulty securing the prosthesis to the talus have been associated with a high failure rate. Outcomes immediately after surgery are good, but the results worsen over time. Other reported complications include infections, inappropriate soft tissue covering and talar injury [14, 15].

In treating this patient, we regarded allografting, endoprosthetic replacement, and treated-bone autografting as difficult to perform; and bone substance and bone strength at the site was insufficient. Although vascularized fibula grafting was the most appropriate treatment, there was a high risk of fracture because the patient was a highly active child. We therefore regarded the bone transport method using external fixation as optimal for treating the bone defect after tumor excision [16]. However, external fixation has problems with patient compliance. The occurrence of pin tract infection following external fixation in patients with malignant tumors makes controlling infections difficult during postoperative chemotherapy [17, 18]. Iodine-coated implants were found to inhibit biofilm formation on the implants and significantly reduce implant-associated infections [19–21]. The preparation of the iodine-coated implant was carried out in the same way as the method we reported earlier [20]. Therefore, iodine-coated implants were used in our patient to prevent pin tract infection.

No evidence of infection was observed in our patient, even during postoperative chemotherapy, suggesting that the iodine coating prevented pin tract infection. Three years after surgery, there was no indication of tumor recurrence, and the patient was able to run without using an orthosis. Future patients, however, may experience growth disorders of affected lower limbs. This reconstruction method may be effective in other patients with tumors of the distal tibia, if pins can be inserted into distal bone fragments after wide excision of malignant bone tumors. However, because autologous bone grafting was necessary during the course, further accumulation of cases is necessary. Application of an iodine coating to these pins may be very effective in preventing pin tract infection.

The bone transport technique has a potential benefit for malignant bone tumors in the distal tibia if pins can be inserted into the distal bone fragments after wide resection. Coating external fixation pins with iodine may prevent pin tract infection.

## Abbreviations

ALP: Alkaline phosphatase
CRP: C-reactive protein
LDH: Lactate dehydrogenase
MRI: Magnetic resonance imaging
MSTS: Musculoskeletal tumor society
